# Qualitative Investigation into the Perception towards Compassionate Parenting among Parents of Autistic Children: Cross-Cultural Comparison between the UK and The Netherlands

**DOI:** 10.3390/healthcare11081199

**Published:** 2023-04-21

**Authors:** Kenneth Curley, Yasuhiro Kotera

**Affiliations:** 1 College of Health, Psychology and Social Care, University of Derby, Derby DE22 1GB, UK; 2 School of Health Sciences, University of Nottingham, Nottingham NG7 2HA, UK

**Keywords:** autism spectrum disorder, parental perceptions, cross cultural study, compassionate parenting, parenting styles, quality of life

## Abstract

Parenting a child with autism spectrum disorder is believed to present challenges that lead to increased levels of stress, as well as a reduction in the quality of the relationship between parent and child. This study aims to investigate parental perceptions toward a compassionate parenting style of parenting to better understand how this style may influence relationships and quality of life in parents. Parents from the United Kingdom (six parents) and the Netherlands (five parents) were invited take part in semi-structured interviews, whereby the data collected were subsequently analysed using thematic analysis. Data from both British and Dutch groups were overall similar to each other. Four themes were identified from the aggregated data: (a) “Parents believe compassionate parenting is important” (parents believed that compassion is an essential element of their parenting style and improves situational outcomes); (b) “Compassionate parenting de-escalates stressful situations” (compassionate parenting reduces stress and improves the quality of life); (c) “High pressure situations as a threat to practice compassion” (challenges and limitations to compassionate parenting style); and (d) “Greater public and professional awareness of autistic behaviours” (the general public and professional services often lack awareness to recognise autistic behaviour traits). Results are consistent with research examining the perceptions of parents of neurotypical children, in that a more compassionate approach to parenting is valued, as it is believed to create a greater connection with the child. Our findings inform researchers and educators as to what parents of children with ASD find useful, important, and worthwhile. Future research needs to investigate how compassionate parenting impacts autistic children’s quality of life.

## 1. Introduction

Autism spectrum disorder (ASD) is described as a pervasive neurodevelopmental disorder, and characterised as impairments in social interaction and communication, accompanied by repetitive behaviours patterns [1]. While these characteristics comprise the core aspects of ASD, the degree to which an individual is affected is believed to differ from person to person. Longitudinal studies suggest that symptoms can often change over time. However, this is thought to depend on factors such as early intervention, situational circumstances, and caregiver engagement [2,3]. 

ASD among children has been increasingly diagnosed in many countries, including the United Kingdom (UK) and the Netherlands. According to recent figures from the World Health Organization (WHO) [4], the global rate for ASD in children is one in 270. In the United Kingdom, this figure is estimated to be one in 100 children [5] and in the Netherlands, this is also suggested to be one in 100 children [6]. Associated with the rise in prevalence of ASD are reports on the challenges associated with parenting a child with this disorder, which seem to appear frequently in research [7,8,9]. 

Emerging evidence indicates that parents of children with ASD, as compared to other child-related disabilities, experience the highest levels of stress [10], and child maladaptive behaviours, such as temper tantrums and aggressive behaviour, are believed to be the main contributing factor for this rise [10]. The effects of stress on family quality of life and, in particular, its effects on the quality of the relationship between parent and child are primary concerns of this study. 

Stress is understood to influence a person physically as well as mentally and is believed to arise when one perceives a situation or its consequences as being beyond a capacity to cope [8,11]. While stress is, to an extent, considered healthy, a number of serious health issues arise when its frequency and intensity increase [12]. Examples of this are high blood pressure, heart disease, heart attacks, asthma, and irritable bowel disease infections [13], while negative mental health can cause burnout, depression, emotional exhaustion, and fatigue [14]. 

A literature review [15] focusing on stress reduction interventions for the parents of children with ASD outlined four theoretical approach options available, behaviour-based, cognitive behaviour-based, humanistic-based, and psychoeducation-based. The review concluded that each examined intervention was reported as affective at reducing levels of stress in participants. The behaviour-based approaches were described as various forms of parenting training that were often derivatives of applied behavioural analysis (ABA) [16]. This consisted of child behaviour management tools that assists parents with modifying specific atypical behaviour believed to be a key cause of parental stress. The cognitive behaviour-based approaches were highlighted as offering parents a multitude of stress reduction tools, including mindfulness and optimism techniques [17]. Humanistic approaches were shown to reduce stress by either relaxing the body as the result of dance movement or using a phenomenological process, such as a written discloser intervention, to express repressed traumatic experiences, which, as a result, over time reduced stress in participants [18]. Finally, the psychoeducational approach was outlined as an information sharing intervention where participants were provided with an ASD informational manual that offers parents insight into ASD as a disorder and the type of behaviour that may be expected from a diagnosed child [19]. As the interventions outlined here, with the exception of psychoeducation, are taught and guided by a practitioner, and therefore come with a cost to facilitate, finding feasible options to reduce this associated cost may prove beneficial for parents. For example, utilising an intrinsic skill that is believed to be inherent to all individuals, namely that of compassion, may ensure that intervention is more accessible, natural, and attractive to those in need.

One option in particular is that of a contemporary cognitive behaviour-based approach that has shown promise in reducing parenting stress, compassion-focused therapy (CFT) [20]. CFT is a therapeutic intervention based on psychological science, attachment theory, and evolutionary psychology, placing key importance on the need for psychological safety for a person to grow and develop [21]. One fundamental concept in CFT is that of the three emotion regulation systems, the soothing system, the treat system, and the drive system, which are believed to interact based on one’s situational perspective. The function of each is as follows: the threat system is engaged when one feels danger is present, the drive system is engaged when one is in search of a goal, and the soothing system is engaged when one is actively soothing and caring for oneself [21]. 

Compassionate parenting has been broadly defined as an attempt by a parent or carer to see things through the eyes of the child and convey how the child is valued and understood [22]. This can be shown when a parent/carer engages and communicates empathically with their child and the sense of the child’s experience is considered with the intention of alleviating any suffering or distress the child might be experiencing [23]. A process where one is actively being kind and caring to one’s self, resulting in an expansion of the soothing system and reduction in suffering and sense of threat [24]. It is little known how parents of children with ASD perceive this style of parenting, which signifies a gap in knowledge needing exploration. 

### 1.1. Parenting Styles

Parenting styles can be defined as approaches that parents utilize to raise their children. According to recent literature [25], there are four core parenting styles, namely authoritative, authoritarian, permissive, and uninvolved. The authoritative style is believed to be the most effective of the four, with parents using this style to exercise warmth and supportive communication while still setting clear rules and expectations. The authoritarian style is characterised as a highly demanding approach where strict rules are reinforced by punishment, and the feelings and opinions of the child are generally not taken into consideration. In contrast to this, the permissive style demands very little in the way of rules and conformance, with warmth and emotional support thought to be the primary focus of the parent. The final parenting style is uninvolved, this approach is nether demanding or emotionally supportive, there is little warmth communicated and any form of parental guidance is absent. According to a quantitative study on parenting styles and their relationship to parenting a child with ASD, findings indicated the highest levels were in authoritative parenting, followed by permissive and authoritarian, respectively. 

While compassionate parenting has recently become a topic of increasing interest, mainly due to the belief that it mends relationships and helps individuals interconnect and promote emotional intelligence [26], there is little known on how parents of children with ASD perceive compassionate parenting as a parenting style. This study aims to fill this knowledge gap by interviewing parents in the UK and the Netherlands to understand their thoughts and feelings on this question. We focused on British and Dutch parents because there is little research comparing these two cultures and as both authors reside in these countries and therefore have first-hand experience of each culture and the lived experience of the service provided as a parent of an autistic child.

### 1.2. How Culture Influences Parenting

According to Chen et al. [27], culture has a significant effect on parental behaviours, beliefs, and values. It is suggested that culture can define parental goals and the different beliefs associated with this can influence child rearing and approaches to discipline. Examples offered by Chen et al. [27] outline how respect and obedience can be a parenting goal in one culture but be of less importance in another, especially when conflicting with goals that support independence and self-expression. These cultural differences lead to parenting styles such as authoritarian or authoritative parenting [27]. 

### 1.3. Study Aims

This study aimed to investigate perceptions towards compassionate parenting among parents of children with ASD and perform a cross-cultural comparison between parents in the UK and the Netherlands. Using semi-structured interviews, we investigated how parents think and feel about the advantages and disadvantages of compassionate parenting, along with their challenges and successes using this style of parenting. Two research questions were established. 

RQ1: How do parents of children with ASD perceive compassionate parenting? 

RQ2: How do these compare cross-culturally between the UK and the Netherlands?

## 2. Materials and Methods

### 2.1. Study Design

A qualitative study with a thematic analysis of semi-structured interviews was employed. All participants agreed to a one-hour interview via video conferencing, which allows for face-to-face contact between the participants and the interviewer. Utilizing such mediums of communication allows for feasible and accessible settings that can create a greater sense of personal contact than through a telephone or email [28,29]. All interviews were audio-recorded (with prior consent) for the transcription (verbatim) and analysis. The participants were a mixture of full-time and part-time stay-at-home carers. The lead researcher was based in the Netherlands. 

### 2.2. Participants

Participants for this study were recruited from social media. An invitation containing the researcher’s contact details was posted on Facebook for people who were interested in making contact and participate in this study. One Facebook group for parents of children with ASD was accessed for recruitment for each country, the UK group consisted of 3000 individuals and the group in the Netherlands comprised 5300 members. All 11 participants that registered for the study satisfied eligibility criteria and were included. This included being a British/Dutch citizen or a resident of 5 years or longer, being a parent of a child diagnosed with autism, being a parent aged 18 or older, and having a good level and understanding of English. The invitation contained a consent form and information explaining why they were being invited to take part in this study and why it is being conducted. Eleven parents contacted the researcher and completed the study to completion, with each parent having at least one child diagnosed with ASD. There were six UK participants: four females and two males. The five participants from the Netherlands included three females and two males. 

### 2.3. Procedure

Ethical approval was granted through the research ethics committee of the university of the lead author, K.C. Once consent was completed, the interviewer and the participant arranged a time to meet online. Semi-structured questions were utilised to obtain participants’ thoughts and experiences. The interview questions consisted of seven open-ended questions in the English language: What do you think or feel about compassionate parenting? How important do you think compassion is in your parenting? Any episode or example where compassion worked in your parenting? When did you feel using compassion was difficult? What made it difficult? What are the positive/negative effects of compassionate parenting for your child, you, and your family? What do you think of the culture or country’s care system for autistic children and awareness of autism? What do you hope for regarding care for autism in the future? The use of semi-structured interviews allows participants express their thoughts and feelings more freely. According Kallio et al., [30] this style of interview creates a relaxed atmosphere which helps elicit more honest responses from participants. Data were analysed using thematic analysis because this analysis affords an element of flexibility and reciprocity between the participant and the interviewer and permits a more casual tone, which is thought to elicit more relaxed and honest responses [31,32].

Online interviews are economical and geographically adaptable [33]. Each interview explored the participants’ perceptions, and the list of questions explored the following lines of enquiry. Throughout the interview, active listening skills and open-ended questions were used to explore the experiences of participants. The researcher conducting the experiments had experience in listening to and facilitating the exploration of experience (counselling and psychotherapy) [34,35]. Throughout the procedure, the lead researcher reported no experiences or incidences of bias that might have led to any form of data interference. Participants only knew the researcher/interviewer’s gender and name before the interview—any political or religious views of the researcher were unknown to the participants. Participation compensation was not offered.

### 2.4. Thematic Analysis

As stated in the procedure, thematic analysis was employed to group similar and repeated thoughts, ideas and experiences under one descriptor. This organised the lengthy data collected from each interview into meaningful concepts that were later followed up for interpretation [36]. The thematic analysis process followed a six-phase approach, as proposed by Braun et al. [37]: Phase 1, familiarisation with the data; Phase 2, generating initial codes; Phase 3, searching for themes; Phase 4, reviewing potential themes; Phase 5, defining and naming themes; and Phase 6, producing the report. The data were read and reread for absorption, followed by initial code labelling. Thirty-three codes were generated (e.g., compassion as a tool in a toolbox, recurring non-conforming behaviours helped by a compassionate parenting style, time constraints, and generational differences causing stressful situations), which were grouped into themes and arranged using mind-mapping techniques for organisation according to class, creating broad topics and issues as possible thematic descriptors [38,39]. 

## 3. Results

Four themes were identified from the aggregated data: (a) “Parents believe compassionate parenting is important” (parents believed that compassion is an essential element of their parenting style and improves situational outcomes); (b) “Compassionate parenting de-escalates stressful situations” (compassionate parenting reduces stress and improves quality of life); (c) “High pressure situations as a threat to practice compassion” (challenges and limitations to compassionate parenting style); and (d) “Greater public and professional awareness of autistic behaviours” (the general public and professional services often lack awareness to recognise autistic behavioural traits). Each of the 32 codes collected were assigned to their respective theme, as can be seen in Table 1. Table 2 presents text excerpts next to their respective themes, and Table 3 displays observed similarities and differences between the two cultures. 

The data analysis of “a view that compassionate parenting is important” revealed a cross-cultural view that compassionate parenting is an important parenting style to practice and a valuable method for promoting the development of empathy and compassion in children. The data extracts of “compassionate parenting improves quality of life” revealed how compassionate parenting practices are believed to improve and maintain the overall quality of life of the family unit. The data extracts of “challenges and limitations to a compassionate parenting style” revealed how life and personal challenges are viewed as limits to a person’s capacity to be compassionate when under certain stresses and pressures. Lastly, the data extracts of “a need for greater awareness, acceptance and support” revealed a cross-cultural view that greater public awareness is needed in recognising autistic behaviour traits. In addition to this, there was a desire for easier access to institutional supports that offer financial funding and possible compassionate parenting training to families of children with ASD. 

### 3.1. Theme 1: A View That Compassionate Parenting Is Important

Each theme will be explored in greater detail now, and examples from the transcription section will be provided to support the decision for each theme title. Theme 1: a view that compassionate parenting is important. All 11 participants stated that they view compassionate parenting as an important parenting style for communicating love, a sense of worth and understanding to their child.

*Participant 3 (UK): “I don’t understand any parenting without compassion, I think compassionate parenting leads to that kind of development, it’s very important.”*

Participants noted the importance of compassionate parenting as a modelling tool for their children. Namely, to develop compassionate and empathic communication skills.

*Participant 11 (NL): “It’s important for anticipating his emotions and trying to understand why he is reacting the way he does. And try to be a step ahead of how he will react and try to understand whenever he reacts in one way or another, why he is behaving this way. What are the triggers that are bringing him to that and of course I will try to avoid those triggers? I tried to figure out if there’s anything I can do to help him and keep him happy.”*

In contrast to the appreciation for a compassionate style, many participants reported that there were situations where practising compassionate parenting did not change or modify their child’s behaviour. For example, when the child was acting out a temper tantrum, practising compassionate parenting did not reduce or stop the behaviour in the moment. Therefore, some participants viewed compassionate parenting as ineffective for stopping maladaptive behaviour when the child is in the grip of emotions such as anger.

*Participant 9 (NL): “The negative part is, there are days that it helps, then there are days it does not help, and there’s nothing you can do about it.”* (This refers to the child experiencing a meltdown or when the child is behaving aggressively and uncontrollably).

### 3.2. Theme 2: Compassionate Parenting Improves Quality of Life

All participants shared examples where compassionate parenting was useful in their parenting. The examples that follow demonstrate how a compassionate parenting style improved the quality of life of each parent, child, and family unit as a whole. The primary factor in achieving an improved quality of life is thought to be the identification of a set of triggers that lead to the child’s emotional aggravation. Therefore, with this information, parents are better able to de-escalate, reduce, and prevent stressful situations from occurring. The result of which increases moments when the child is present in a calmer state, enriching the quality-of-life moment to moment.

*Participant 2 (UK): “When I’m brushing my daughter’s hair, I have to have some understanding that it hurts her, it shouldn’t but it does and it upsets her, so you just have to be gentle and take time and try not to be in a rush. So, I am compassionate in that way but when she is having a meltdown, you have to really realise that sometimes you just need to sit with her and hold her tight and stay with her and say nothing, what’s more important.”* This extract demonstrates how the compassionate understanding of the child led to de-escalating a meltdown and created an opportunity for the deep connection between parent and child.

*Participant 6 (UK): “So, we’re going to the supermarket, and I thought this is great, he’s enjoying it and he’s having a great time. Then he started to say, I want this, I want that, and I’m thinking oh no, how do I say no here. I want to give him everything, but I can’t give him everything every time. But I can’t say no, so that was really difficult. I ended up having to be compassionate and learn how to go around saying no.”* This extract shows how this parent felt that using compassionate parenting helped with managing the onset of a possible meltdown.

*Participant 9 (NL): “So, for example, you could just sit with her and you get a punch in the face, but you have no idea why and she is so strong and you feel flabbergasted and the first thing you want to do is ask what did I do to deserve this? Then I say, you hurt your dad a lot and I’ve no idea what I did. Then genuinely she says I’m sorry dad, I’m sorry that I did it, I’m sorry I hit you.”* This extract demonstrates how a violent act toward the parent was met with kindness and compassion, which led to the child eventually regretting and apologising for the action.

### 3.3. Theme 3: Challenges and Limitations

Theme 3: challenges and limitations. All participants described common and unique challenges to practising compassionate parenting. These varied from work and home demands, such as a second or third child, time constraints, extreme meltdowns, low energy, the pressure to conform to parenting norms in public, everyday stresses, and new and unexpected situations or experiences.

*Participant 7 (NL): “I have my own company and I am busy not only in work but outside work and you’re thinking fast, and I want to get things done fast, but the kid doesn’t understand this.”*

*Participant 8 (NL): “When there isn’t enough time then you know I don’t have the patience for it. So I think it’s important but it’s only possible to do it in my own time, I have to really think about compassionate parenting when it’s hard (at difficult times) but not in the moment (when everything calms down) I think maybe I have to chill out first and figure out how to sort out this problem and that’s probably the hardest part.”*

*Participant 10 (NL): “When you have a second child and one child is crying and crying and the other is getting depressed and crying as well. Then I learned, first look after the toddler and then the baby. That was difficult and sometimes you might feel like you’re a bad parent because one is crying and crying and you’re attending the toddler first and then the baby because he is more difficult to handle.”*

### 3.4. Theme 4: A Need for Greater Awareness, Acceptance, and Support

All participants claimed they believed there is a lack of public awareness for recognising autistic behaviour traits. For example, they believed that while the public is aware of a disorder called autism, they do not recognise autistic signs and behaviours, such as meltdowns associated with emotional regulation, preferences for social isolation, or repetitive behaviours that are thought to relax and soothe a child with ASD.

*Participant 3 (UK): “I’d like to see more awareness, more open-mindedness, and not be so stuck in a square box that people accept. You know there are a lot of parents out there that do not have the knowledge. It’s really disappointing, people get so tired and exhausted that they don’t have a husband or someone to support them.”*

Many parents shared how they had to struggle and fight with their local authorities to obtain access to the supports their child needed. For example, one parent shared how they had to take legal proceedings against their local authority before they finally received the necessary support. In other cases, parents pay extremely high fees to fund special education for their autistic children which would normally be funded by their state.

*Participant 1 (UK): “Local governance, there is a lot of fighting, there is no appreciation for the costs and the effort involved to put any program in place for ASD children. I don’t know if it’s the budget cuts or what but getting help is quiet. The government won’t fund ABA support.”*

In another case, a participant shared that he believes that without the support his child and family receive from the state, he and his wife would no longer be together. He named the stresses and demands of parenting a child with ASD as a great challenge and without receiving support, it would be too much for them.

### 3.5. Cross-Cultural Analysis Summary (RQ2)

Upon analysing interviews with both UK and Dutch parents of children with ASD on the topic of compassionate parenting, various parenting styles alongside compassion were observed (see Table 3). The most prevalent style was authoritative. Within Theme 1, there were similarities whereby compassionate parenting is viewed as an essential communication style for demonstrating love, worth, and understanding to their child. Parents from both countries noted the importance of using compassionate parenting as a modelling tool for teaching empathic communication skills. All participants believed that compassionate parenting improved and maintained the overall quality of life for their families by recognizing triggers to stressful situations and avoiding them through empathy and preparation. However, many participants also reported challenges in practicing compassionate parenting—this ranged from work and home demands to extreme meltdowns, limiting the capacity to exercise compassion under pressure and stress.

There were cultural differences observed among the participants. UK participants perceived a lack of awareness of autism, while in the Netherlands, there was a sense that society had an awareness of autism but did not recognize autistic behaviour or know how to engage it. Many UK parents felt pressure to abandon compassionate parenting in public to avoid being judged as bad parents if they did not exercise authoritative behaviour with their child during a meltdown. In the Netherlands, a similar belief existed, although the pressure to exercise an authoritative appearance seemed more intrinsically driven than reactive to societal criticisms. Additionally, regarding the difficulties and effort in gaining access to support systems in the UK, it should be noted that UK parents were confronted with difficulties obtaining financial support and specialized care. Although Dutch parents encountered some difficulties in accessing support systems, they generally viewed the support systems available as satisfactory.

## 4. Discussion

This qualitative investigation into the perception towards compassionate parenting among parents of children with ASD a cross-cultural comparison between the UK and the Netherlands involved 11 participants, all of whom were the parents of at least one child diagnosed with autism spectrum disorder. Four themes emerged from the analysis of the data: (Theme 1) a view that compassionate parenting is important; (Theme 2) compassionate parenting improves quality of life; (Theme 3) challenges and limitations; and (Theme 4) a need for greater awareness, acceptance, and support. 

All participants stated that they viewed compassionate parenting as an important parenting style for communicating love, understanding, and developing compassionate communication in their child, with a few expanding on this importance and expressing their hopes that by practising compassionate parenting, their child might develop a compassionate and empathic mindset in the future. This is consistent with past research that indicated that some parents value a more compassionate approach to parenting, as it was believed a greater connection with the child could be attained [40]. Moreover, Breen [41], highlighted that the parental modelling of empathic communication is an important component for child psychosocial development, which was identified earlier in the introduction as a specific impairment of children with ASD. 

It is important to note here that although parents viewed compassionate parenting as an important parenting style, many indicated that there were situations where practising compassionate parenting was either not practically possible or did not bring about an immediate change in the child’s behaviour. These findings will be explored further when examining Theme 3 in more detail, regarding the challenges and limitations, with particular attention given to time constraints and other demands. 

Many participants described that they felt naturally drawn towards responding compassionately to their child because they were autistic. This was a consistent theme for both parents in the UK and in the Netherlands, reinforcing the belief that the experience of parenting a child with ASD has the potential to naturally build compassion in parents. Conti [40] suggests that the practice of compassionate parenting often develops further into a sense of well-being and meaning of life.

All participants shared examples of how practising compassionate parenting worked for them. In those examples, it can be seen how a compassionate parenting style improved the quality of life of the parent, the child, and the family unit as a whole. The key factors for improvement are believed to be the understanding shown by parents and their skills in recognising the signs that trigger emotional irritation in the child and thereby taking evasive measures that prevent stressful or tense situations from happening. This is consistent with findings in a study that concluded that compassionate parenting promotes positive outcomes associated with caring and empathic communication [42]. To further support this, a similar report investigating the benefits of offering a compassionate family care service highlighted a number of benefits which included: decreased parental stress, decreased anxiety and depression, increased confidence in caregiving skills, greater development of early interaction skills, and improved parent satisfaction [43].

All participants described common and unique challenges to exercising compassionate parenting regularly. These vary from work and home demands, such as a second or third child; time constraints; extreme meltdowns; low energy; a pressure to conform to traditional parenting norms in public; and everyday stresses, along with sudden or unexpected situations and experiences. It appears evident that these demands weigh heavy on parents of children with ASD. So much so that the ability to exercise compassion seems almost too much to consider certain conditions. This is consistent with research that correlates to the increased levels of stress of parenting a child with ASD, generally due to the experience of maladaptive or aggressive behaviour often reported by parents of children with ASD [10].

Participants in both the UK and the Netherlands claimed that they believed there was a lack of public awareness in recognising the core behaviours of autistic individuals. For example, they believed that while the public in their country is aware of a condition/disorder called ASD, they do not recognise many autistic signs and behaviours, for example, meltdowns associated with emotional regulation or a preference for social isolation to comfort their social anxiety. This cross-cultural view is consistent with a recent study into improving ASD awareness, which revealed how respondents wished for better public awareness and noted that education was paramount in attaining greater understanding of ASD [44]. In addition to this, participants from the UK expressed a view of dissatisfaction for a perceived lack of knowledge in certain professional settings, specifically primary teachers and some general practitioners. In the many views shared, participants believed this was partially due to insufficient training in ASD recognition and managing an individual displaying ASD traits. 

With regard to local authority and ASD support, many parents shared examples of the resistance they encountered in their efforts to attain the necessary support for their child. This theme was shared across the UK and the Netherlands, with greater resistance being reported in the UK. The examples reported describe how some parents had to fight with their local authorities or schools to obtain access to the support their child needed. For example, one parent shared how they had to take legal proceedings against their local authority before they finally received the support their child required. This is consistent with a UK government report outlining the critical state of obstacles faced by parents of children with ASD [45]. In one instance, a participant reported spending extremely high school fees themselves to fund special education, such as that given by Applied Behavioural Analysis (ABA) schools, because they could not obtain local funding. 

As previously explored, there are added challenges and increased levels of stress being reported by parents of children with ASD. However, little attention has been given to how this stress may affect the relationship between the carers or parents of children with ASD. One participant in the Netherlands shared how he believed that without the support that his child and family received from the state, he and his wife would no longer be together. He identified the stresses and demands of parenting a child on the spectrum as the most difficult element pressing on them both, and that without the support they received, it would be too much for them both to handle. Some examples of the support they receive are financial support that enables their child to attend a weekend camp and a specialised school. It may be noteworthy to mention here the findings highlighted by Bluth et al. [46], which outlined how empathic and caring communication skills practised between parents of children with ASD can lead to reduced levels of stress and conflict and promote satisfying and supportive relationships. 

### 4.1. Limitations

Limitations found in this study include the small sample size, namely *n* = 11. This may have implications for transferability to a greater population; however, this number seems justified and consistent with qualitative sampling size standards as concluded by a systematic review of empirical tests [47]. Another limitation found is the absence of a double coder to confirm the validity and possibility of any bias. All authors reported no conflicts of interests or difficulties with neutrality throughout the study. 

### 4.2. Implications

The implications of our findings indicate views that greater awareness of autism is needed in public, academic, and professional settings, such as teachers and general practitioners. This could include information on how to recognise and engage with an individual with ASD and would be expected to lead to greater awareness of autism, which could lead to a national push towards autism inclusion. 

### 4.3. Further Research

Future research exploring teachers’ and general practitioners’ awareness of autism may shed light on the parent perception that this is often lacking. A literature review followed by a mixed method study combining interviews and questionnaires may provide important information from a professional perspective. The results could reveal a gap in services and lead to additional training in these settings. In addition to this, many parent interviews highlighted the enthusiasm for a compassionate parenting skills training. While there was a belief that compassion felt like a natural response to parenting a child with ASD, many challenges to practicing it were shared. Future research, such as a randomised control trial investigation into the effectiveness of compassion training for parents of children with ASD may show positive results in increasing compassionate parenting skills and practice. As outlined in the introduction, the benefits of this would be revealed through the empathic communication of a parent with their child and the alleviation of suffering or distress [23], thereby increasing quality of life as a result. 

## 5. Conclusions

This investigation into the perception towards compassionate parenting among parents of autistic children has been explored and has revealed the thoughts and feelings of parents from across the UK and the Netherlands. The four themes that emerged, namely (a) a view that compassionate parenting is important, (b) that compassionate parenting improves quality of life, (c) challenges and limitations to a compassionate parenting style, and (d) a need for greater awareness, acceptance, and support, outline the range of thoughts and feelings that comprise the perceptions of parents toward compassionate parenting, and in so doing, has highlighted how beneficial this style of parenting can be at reducing parental stress and improving the relationship between parent and child and ultimately quality of life. The findings of this study have identified a number of cross-cultural challenges faced by parents and revealed a shared view that there is a lack of both public and professional awareness in recognising ASD and difficulties in practising compassion in certain situations. How to overcome these challenges could be the focus of future research, specifically in the area of support and when compassion is perceived to be too difficult to practise. Our findings extend the body of research investigating compassionate parenting among parents of children with ASD and advocates that a compassionate parenting style improves quality of life, promotes positive social skills, and creates a deeper connection between carer and child. 

## Figures and Tables

**Table 1:** Themes and codes.

Theme 1: (A View that Compassionate Parenting Is Important)	Theme 2: (Compassionate Parenting Improves Quality of Life)	Theme 3: (Challenges and Limitations to a Compassionate Parenting Style)	Theme 4: (A Need for Greater Awareness, Acceptance, and Support)
A critical view of compassionate parenting Compassion as a tool in a toolbox Manipulation of compassion	An example demonstrating when compassion worked	Concerns of child manipulating the situation	A sense of general autism awareness
	Creates calm opportunities for development	Extreme meltdowns	A sense of social inclusion
	Opportunity for the child to learn and model compassion	In public, pressure on parents to follow cultural norms	An understanding society
Parenting can be difficult and stressful	Recurring non-conforming behaviours helped by a compassionate parenting style	Low energy	Critical society
Positive view of compassion	Reduces stress and de-escalates tension	New experiences	Generational differences causing stressful situations
Financial costs with outside assistance are high	Supports mental preparation	Other demands	Governmental funding cuts
Stresses of parenting ASD children		Stress	Hope for the future
Supporting ASD characteristics		Time constraints	Institutional support
			Needed to fight for support
			Parents funding autism child development plan

**Table 2:** Responses to questions.

No	Theme	Example Participant Excerpt
1	Theme 1: (A view that compassionate parenting is important) RQ1	*“I think it’s extremely important to do, I don’t know how to do it any other way, the positives are she is less stressed, less anxious and she feels understood. I’m hoping to give her the resilience that she needs with compassionate parenting to be able to be self-aware. If we can have that empathy or compassion, then the person will feel heard and valued. I think it will rub off at some stage and then they will show compassion. A positive thing is that there is more harmony in the family. Everything runs smoothly and everything is functioning. Everything is fine and I think my child is thinking that mummy understands me, and I have a voice and I can have my opinion and I am a person.”*
2	Theme 2: RQ1 (compassionate parenting improves quality of life)	*“I don’t know about my child but I’m less angry, less annoyed, and more understanding. As a household we’re more emotionally intelligent. If I’m going to react badly to him for not communicating properly, then that’s going to put him down even more and have a worse quality of life. I learned from past events that understanding your child makes a difference because life experiences are stressful. Listening to your child is very important for the standard of living. Parents need to listen to the child; you cannot rely on someone else to do it.”*
3	Theme 3: RQ1 (challenges and limitations to a compassionate parenting style)	*“When there isn’t enough time then you know I don’t have the patience for it. So I think it’s important but it’s only possible to do it in my own time, I have to really think about compassionate parenting when it’s hard (at difficult times) but not in the moment (when everything calms down) I think maybe I have to chill out first and figure out how to sort out this problem and that’s probably the hardest part. So, the point is, the decision is how tired I am. If I’m too tired to deal with this, then another time is fine. I have my own company and I am busy not only in work but outside work and you’re thinking fast, and I want to get things done fast, but the kid doesn’t understand this.”*
4	Theme 4: RQ1 (a need for greater awareness, acceptance, and support)	*“They’re very aware that autism exists here in the Netherlands, but they basically don’t know how to recognise autism, they know about this but they don’t know how to recognise it. Here in Holland the government is making it more difficult to find resources, but my wife and I are educated enough to find a way, but you hear a lot of parents that sometimes have fewer capabilities to find access to resources, to find support. And I think for these parents’ life can be a real living hell because they have to ask for everything. You have to find everything which used to be a lot easier before in the past but now it’s getting very difficult. We had to fight ourselves. If we didn’t even have half the rights of what we have now for sure my wife and I would be divorced. I’m sure that it’s scary that a lot of parents out there are in that situation.”*

**Table 3:** Comparison between the UK and the NL.

No	Theme	UK Observations *n* = 6	NL Observations *n* = 5
1	Theme 1: RQ2 (a view that compassionate parenting is important)	Compassionate parenting utilised in combination with permissive and authoritative styles. Compassion viewed as a very important aspect of parenting.	Compassionate parenting utilised in combination with authoritarian and authoritative styles. Compassion viewed as a very important aspect of parenting.
2	Theme 2: RQ2 (compassionate parenting improves quality of life)	A view that children respond better to compassionate communication. Compassionate mindset leads to being better prepared, leading to the avoidance of irritation triggers. A view that compassion has positive effects on emotional state of the family unit.	A view that compassion improves child behaviour and interpersonal relationships. Compassionate mindset leads to being better prepared, leading to the avoidance of irritation triggers. A view that compassion has positive effects on emotional state of the family unit.
3	Theme 3: RQ2 (challenges and Limitations to a compassionate parenting style)	A view that social stigma makes it difficult to practice companionate parenting in public for not disciplining their child when having a meltdown. Fatigue and frustrations and lead to harsh reactions instead of compassion. Time demands and daily pressures make is difficult to be compassionate	A sense of needing to practice an authoritative parenting style in some public settings. Fatigue and frustrations and lead to harsh reactions instead of compassion. Time demands and daily pressures make is difficult to be compassionate.
4	Theme 4: RQ2 (a need for greater awareness, acceptance, and support)	Perception that a lack of awareness of autism exists in society. Access to support frustrating and difficult. A view that specialised support is missing.	Perception that society is aware and accepting of autism, however engagement lacking. Access to support straightforward however becoming more difficult. A view that specialised support is available but limited variety.

## Data Availability

The data reported in this article can be found in the cited research studies included and referenced. Participant data will remain confidential.
